# Supplementary material for: High Tg/HDL-Cholesterol Ratio Highlights a Higher Risk of Metabolic Syndrome in Children and Adolescents with Severe Obesity
Source: J Clin Med. 2022 Aug 1;11(15):4488. doi: 10.3390/jcm11154488 (PMC9369869; doi:10.3390/jcm11154488)
Supplement: Supplementary file 1 [file jcm-11-04488-s001.zip › jcm-1771359-supplementary.pdf]

Supplementary Table S1: ROC Contrast Estimation and Testing Results by Row among BMI, BMI-SDS, TMI, WtHR, TG HDL, CMI, VAI.

**Contrast**	**Estimate**	**Standard**	**95% Wald**		**Chi-Square**	**Pr > ChiSq**
		**Error**	**Confidence Limits**			
**WtHR - VAI**	-0.293	0.030	-0.352	-0.234	93.883	<0.0001
**TMI - VAI**	-0.301	0.032	-0.363	-0.240	91.716	<0.0001
**TG/HDL - VAI**	-0.004	0.0031	-0.010	0.002	1.423	0.2329
**CMI - VAI**	-0.007	0.005	-0.016	0.002	2.546	0.1106
**BMI - VAI**	-0.222	0.030	-0.281	-0.163	54.002	<0.0001
**BMI SDS - VAI**	-0.279	0.031	-0.340	-0.218	80.454	<0.0001

**Contrast**	**Estimate**	**Standard**	**95% Wald**		**Chi-Square**	**Pr > ChiSq**
		**Error**	**Confidence Limits**			
**VAI – TG/HDL**	0.004	0.003	-0.002	0.010	1.423	0.2329
**WtHR – TG/HDL**	-0.289	0.031	-0.350	-0.228	86.853	<0.0001
**TMI – TG/HDL**	-0.298	0.031	-0.359	-0.237	91.37	<0.0001
**CMI – TG/HDL**	-0.004	0.005	-0.013	0.006	0.564	0.4525
**BMI – TG/HDL**	-0.218	0.030	-0.278	-0.159	52.155	<0.0001
**BMI SDS – TG/HDL**	-0.275	0.031	-0.335	-0.214	79.271	<0.0001

**Contrast**	**Estimate**	**Standard**	**95% Wald**		**Chi-Square**	**Pr > ChiSq**
		**Error**	**Confidence Limits**			
**VAI - CMI**	0.007	0.005	-0.002	0.016	2.546	0.1106
**WtHR - CMI**	-0.285	0.028	-0.340	-0.231	103.979	<0.0001
**TMI - CMI**	-0.294	0.029	-0.351	-0.238	103.97	<0.0001
**TG/HDL - CMI**	0.004	0.005	-0.006	0.013	0.564	0.4525
**BMI - CMI**	-0.215	0.028	-0.270	-0.159	57.417	<0.0001
**BMI SDS - CMI**	-0.271	0.029	-0.328	-0.215	89.381	<0.0001

Abbreviations: BMI: Body Mass Index; BMI SDS: BMI standard deviation score; TMI: Tri-Ponderal Mass Index; WtHR: Waist-to-Height ratio; TG/HDL: triglyceride to high density-lipoprotein cholesterol ratio; CMI: Cardiometabolic Index; VAI: Visceral Adiposity Index.
